# APOE4 rat model of Alzheimer’s disease: sex differences, genetic risk and diet

**DOI:** 10.1186/s12868-024-00901-z

**Published:** 2024-11-06

**Authors:** Bradley Colarusso, Richard Ortiz, Julian Yeboah, Arnold Chang, Megha Gupta, Praveen Kulkarni, Craig F. Ferris

**Affiliations:** 1 Center for Translational NeuroImaging, Northeastern University, Boston, MA USA; 2 Department of Psychology, Northern Illinois University, DeKalb, IL 60115 USA; 3 Departments of Psychology and Pharmaceutical Sciences, Northeastern University, 125 NI Hall, 360 Huntington Ave, Boston, MA 02115-5000 USA

**Keywords:** Diffusion weighted imaging, Functional connectivity, MRI, High fat/high sucrose diet, Graph theory, Sex difference

## Abstract

The strongest genetic risk factor for Alzheimer’s disease (AD) is the ε4 allele of apolipoprotein E (*ApoE ε4*). A high fat diet also adds to the risk of dementia and AD. In addition, there are sex differences as women carriers have a higher risk of an earlier onset and rapid decline in memory than men. The present study looked at the effect of the genetic risk of *ApoE ε4* together with a high fat/high sucrose diet (HFD/HSD) on brain function in male and female rats using magnetic resonance imaging. We hypothesized female carriers would present with deficits in cognitive behavior together with changes in functional connectivity as compared to male carriers. Four-month-old wildtype and human *ApoE ε4* knock-in (TGRA8960), male and female Sprague Dawley rats were put on a HFD/HSD for four months. Afterwards they were imaged for changes in function using resting state BOLD functional connectivity. Images were registered to, and analyzed, using a 3D MRI rat atlas providing site-specific data on 173 different brain areas. Resting state functional connectivity showed male wildtype had greater connectivity between areas involved in feeding and metabolism while there were no differences between female and male carriers and wildtype females. The data were unexpected. The genetic risk was overshadowed by the diet. Male wildtype rats were most sensitive to the HFD/HSD presenting with a deficit in cognitive performance with enhanced functional connectivity in neural circuitry associated with food consumption and metabolism.

**Supplementary Information:**

The online version contains supplementary material available at 10.1186/s12868-024-00901-z.

## Introduction

Alzheimer’s disease (AD) is the primary contributor to dementia among older individuals, impacting more than 35 million individuals globally. Age stands out as the most influential factor in developing AD, as the likelihood of experiencing the disease increases twofold every five years after reaching the age of 65. Another notable risk factor, ranking second in importance, is the apolipoprotein E (*ApoE*) *ε4* allele [1]. A majority of AD patients carry at least one of the two *ApoE ε4* alleles with women having a greater risk for future problems [2]. Indeed, there are several clinical studies on *ApoE ε4* carriers reporting sex differences in cognition with aging. Females *ApoE ε4* carriers are more likely to develop Alzheimer’s disease than male carriers [3–5] and females have a faster rate in declining memory as compared to males [6–8]. *ApoE ε4*, primarily synthesized by glial cells, is involved in lipid metabolism and cholesterol transport [9–11].

Dysregulation in energy utilization associated with metabolic homeostasis that occurs with aging is a contributing factor to the pathophysiology of AD [12]. Age brings a gradual disruption in metabolism, something enhanced by obesity. Indeed, obesity caused by dietary habits is widely acknowledged as a significant contributor to the development and physiological mechanisms of AD [13–15]. Preclinical studies show the “Western” diet, composed of high fat and sugar contributes to this pathology [16–19]. Disease progression in rodent models of AD is exacerbated when a genetic background of *ApoE ε4* is combined with the protracted consumption of a “Western” diet combining different levels and types of fat and sugar [18, 20, 21]. This model combining *ApoE ε4* plus HFD/HSD was used to develop sensors to analyze exhaled gas for small volatile lipids associated with AD [22] and image progression of neurovascular dysfunction with aging [23].

In the present study we hypothesized prolonged exposure to HFD/HSD would exacerbate disease progression in the human *ApoE ε4* knock-in (TGRA8960) rat. In a previous study, we reported 6-month-old male *ApoE ε4* knock-in rats, present with altered gray matter microarchitecture in the sensorimotor and limbic cortices with a decrease in cognitive function; however, 6-month-old female carriers showed little changes in water diffusivity and cognitive behavior but did show enhanced functional connectivity [24]. In this study, looking at the added risk of a HFD/HSD there was no evidence of cognitive dysfunction or functional connectivity in female or male *ApoE ε4* carriers. Instead, male wild-type rats were most affected by the HFD/HSD.

## Methods

### Animals

Male wildtype (*n* = 4), female wildtype (*n* = 5) and human *ApoE ε4* knock-in (TGRA8960) male (*n* = 5) and female (*n* = 6) Sprague Dawley rats, ca. four months of age, were a gift from Inotiv (West Lafayette, Indiana, USA). All genotypes were studied on high fat, high sucrose diet. Rats were placed in Plexiglas cages, with one or two rats per cage, and were kept in a room with a temperature range of 22–24 °C and a 12-hour light-dark cycle (lights on at 07:00 a.m.). They had free access to food and water throughout the study. Following a week of acclimatization to the animal quarters, all rats were fed a high-fat, high-sucrose diet (HFD/HSD) for a duration of 120 days before undergoing testing, at which time they were approximately 240 days old. The specific diet used (TD.88137) was obtained from Envigo (South Easton, MA) and had the following composition by weight: 48.5% carbohydrates (with 34% of it being sucrose), 21.2% fat, 17.3% protein, and 0.2% cholesterol. Shown in Supplementary Fig S1 are the change in body weights (mean ± SD) for each of the experimental groups collected weekly over a ten-week period. The acquisition and welfare of all rats followed the guidelines outlined in the NIH Guide for the Care and Use of Laboratory Animals. The methods and procedures described in this study were approved in advance by the Northeastern University Institutional Animal Care and Use Committee, under protocol number 20-0627R. Northeastern University’s animal care and use program and housing facilities have received full accreditation from AAALAC, International. The protocols employed in this research adhered to the ARRIVE guidelines, which provide recommendations for reporting in vivo experiments in animal research [25]. Animals were monitored daily over the duration of the study for general health, food, and water consumption. A 15% loss in body weight was set as a humane endpoint. If animals reached a humane endpoint they were euthanized with carbon monoxide followed by thoracotomy.

### Barnes maze

The Barnes Maze has been utilized to evaluate spatial learning and memory in different rodent models. A description of the assay as performed in our lab has been published [26, 27]. In brief, the maze consists of a circular platform with 18 escape holes located around its perimeter. Beneath the platform, there was a removable goal box, positioned consistently across all trials. Before each trial, the rat was placed inside the goal box for 1 min and then placed under a covered container at the center of the platform for 30 s. The container was then lifted to start the trial. During the acquisition trials, if the animals were unable to locate the goal box within the 4-minute test period, they were gently guided into the goal box and allowed to remain there for 1 min. Subsequently, they were returned to their home cages between the three trials conducted each day over a span of four days. In evaluating the acquisition trials, all animals were assessed based on parameters such as goal box latency (the time taken for animals to enter the goal box), errors (defined as instances where animals investigated any non-goal box hole within 2 cm with their head directed towards the hole), error duration (the duration spent exploring non-goal box holes within 2 cm), and the distance traveled before entering the goal box. All animal behavior was recorded on video and analyzed for the time it took to find the goal box. The analysis was conducted manually by experimenters who were unaware of the treatment condition, and the results were further confirmed using ANY-maze^®^ software for automated scoring (Stoelting, Wood Dale, IL, USA).

### Novel object preference (NOP) test

NOP consisted of one acclimation day (habituation), one day of familiarization, and a third day of testing for novel object recognition. The box and objects were cleaned with 70% isopropyl alcohol between each mouse exposure to eliminate olfactory cues. At the start of the study, each mouse was placed in an empty 1764 cm [2] Plexiglas box for 3 min each for habituation. There were two phases that each mouse had to go through after habituation: the Familiar Phase and the Novel Phase. 24 h after habituation, the mice were placed in the same box with two identical objects for 5 min for familiarization. Two objects with different size, color, and texture were used for the NOP test. Half of each testing group was familiarized with an identical set of objects. 18 h later, for the Novel Phase, the mice spent another 5 min in the box with one familiar object and one novel object. The Novel Phase was filmed and uploaded to ANY-Maze software for tracking and analysis. Recorded measures included total time spent investigating the novel object, total time investigating familiar object, number of investigations of each object, and discrimination between objects.

Investigation ratios (IR = time spent investigating the novel object/ time spent investigating both objects) were assessed using single-sample, two-tailed t-tests, and performance was compared to chance (i.e., IR = 0.5). An investigation ratio significantly greater than 0.5 indicates that the mice were spending more time with the novel object. Conversely, a ratio significantly smaller than chance was used as an index of a preference for the familiar object. Analysis was performed with GraphPad Prism.

### Neuroimaging

Imaging sessions were performed using a Bruker Biospec 7.0T/20-cm USR horizontal magnet (Bruker, Billerica, MA, USA) equipped with a 2 T/m magnetic field gradient insert (12 cm inner diameter) that had a rise time of 120 µs. The radio frequency signals were transmitted and received through a quadrature volume coil integrated into the animal restrainer (Ekam Imaging, Boston MA, USA) [28]. The animal restrainer was designed with a padded head support, eliminating the need for ear bars, thereby reducing animal discomfort and minimizing motion artifacts. All rats were imaged under 1–2% isoflurane anesthesia, maintaining a respiratory rate of 40–50 breaths per minute. At the start of each imaging session, a high-resolution anatomical dataset was acquired using the Rapid Acquisition, Relaxation Enhanced (RARE) pulse sequence with the following parameters: 35 slices with a thickness of 0.7 mm, a field of view (FOV) of 3 cm, matrix size of 256 × 256, a repetition time (TR) of 3900 msec, an effective echo time (TE) of 48 msec, a number of excitations (NEX) of 3, and an acquisition time of 6 min and 14 s.

### Resting state functional connectivity

Scans were acquired using a spin-echo triple-shot EPI sequence with the following imaging parameters: matrix size of 96 × 96 × 20 (height x width x depth), a repetition time (TR) of 1000 msec, an echo time (TE) of 15 msec, voxel size of 0.312 × 0.312 × 1.2 mm, slice thickness of 1.2 mm, 200 repetitions, and a total acquisition time of 10 min. Preprocessing steps involved the combined use of Analysis of Functional NeuroImages (AFNI_17.1.12), FMRIB Software Library (FSL, v5.0.9), Deformable Registration via Attribute Matching and Mutual-Saliency Weighting (DRAMMS 1.4.1), and MATLAB software. Brain tissue masks were manually drawn using 3DSlicer and applied for skull-stripping. Motion outliers and spikes were identified and regressed out, followed by slice timing correction. Head motion correction was performed using the first volume as a reference, and normalization was achieved through affine registration to the 3D MRI Rat Brain Atlas. A total of 173 annotated brain regions from the atlas were used for segmentation. Subsequent steps included quality assurance, band-pass filtering (0.01 Hz ~ 0.1 Hz) to reduce drift and noise, detrending, spatial smoothing (full width at half maximum = 0.8 mm), and nuisance regression using motion outliers, motion parameters, mean white matter, and cerebrospinal fluid time series as regressors.

The region-to-region functional connectivity analysis involved calculating correlations in spontaneous BOLD fluctuations. Nodes represented specific brain regions of interest (ROIs), and edges represented connections between regions. Voxel time series data were averaged within each node based on residual images obtained from nuisance regression. Pearson’s correlation coefficients were computed across all pairs of nodes (14535 pairs) for each subject in all three groups to assess interregional temporal correlations. The resulting correlation values (ranging from − 1 to 1) were transformed using Fisher’s Z transform. Symmetric connectivity matrices of size 166 × 166 were constructed, with each entry representing the strength of an edge. Group-level analysis was conducted to examine functional connectivity in the experimental groups. Z-score matrices from one-group t-tests were subjected to K-nearest neighbors clustering to identify node clusters and resting state networks. A threshold of |Z|=2.3 was applied to remove weak or spurious connections between nodes for visualization purposes.

### Resting state BOLD functional connectivity analysis

#### Degree centrality

The network analysis was conducted using Gephi, which is an open-source software for network analysis and visualization (Bastian et al., 2009). The symmetric connectivity matrices of both the CBD and vehicle groups, consisting of absolute values, were imported into Gephi, and the edges were treated as undirected networks. Degree centrality analysis was employed to measure the number of connections each node had within the overall network. The formula for degree centrality (C_D) is defined as the sum of Aij, which represents the number of edges between nodes i and j, with n representing the number of rows in the adjacency matrix A.

$$\:{\text{C}}_{\text{D}}\left(\text{j}\right)=\sum_{\text{j}=1}^{\text{n}}{\text{A}}_{\text{i}\text{j}}$$

#### Statistics

Statistical analysis for the graph theory analysis was performed using GraphPad Prism version 9.0.0 (86) software from GraphPad Software, San Diego, California, USA (www.graphpad.com). Normality tests, such as Shapiro-Wilk’s test, were conducted to assess the normality assumption between group subregions. If the p-values for subregion degree centrality were greater than 0.05, it was assumed that the data followed a normal distribution. In such cases, paired t-tests were used to compare the degree centrality between the CBD and vehicle groups in different subregions. If there was evidence against the normality assumption, a nonparametric Wilcoxon signed-rank (WSR) test was performed instead.

## Results

### Cognition

**Fig. 1:**
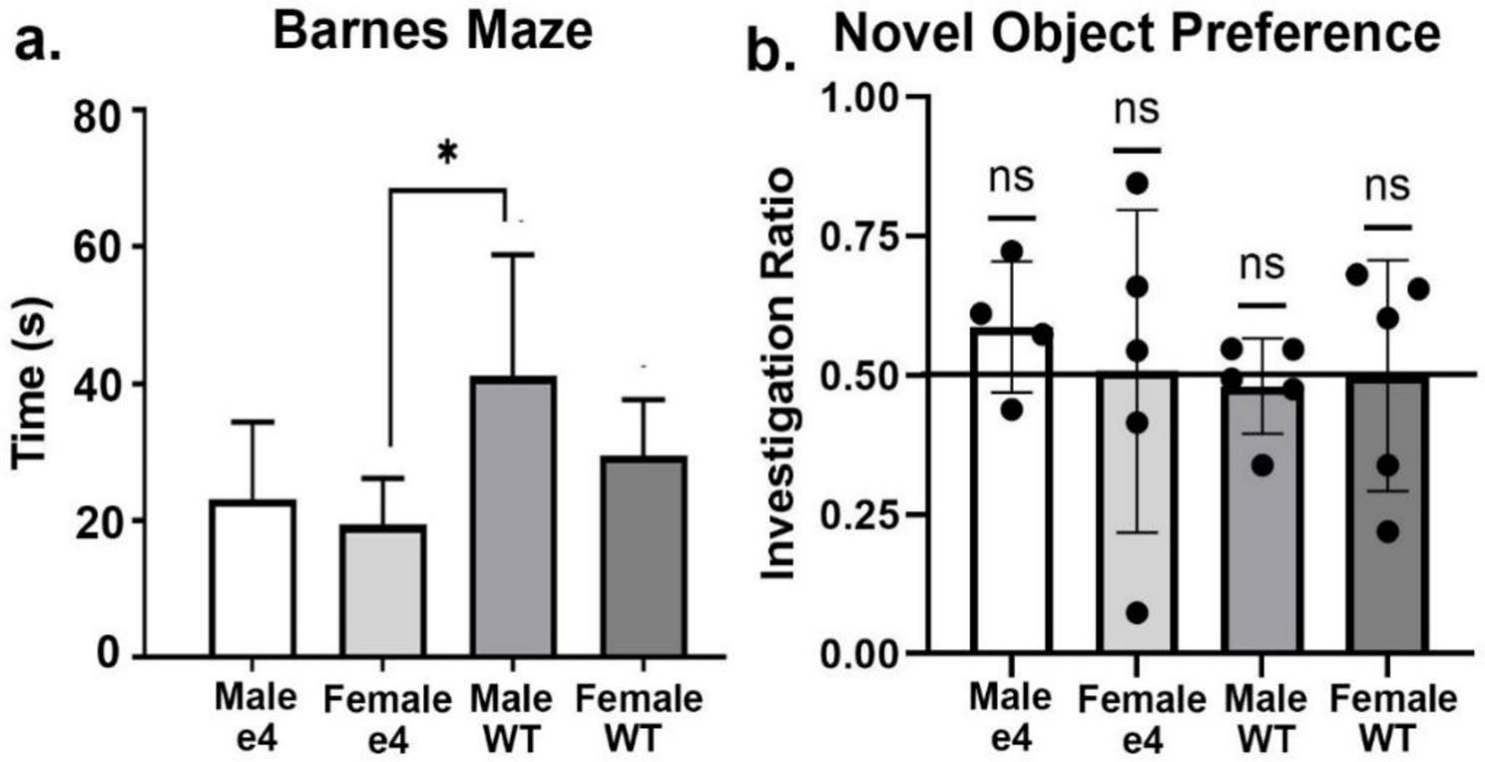
Behavioral tests. Shown in (**a**) is the time in sec (mean ± SD) or latency to find the goal box. Male wildtype (M/WT) were significantly slower than female *ApoE ε4* (F/e4). Data from NOP are shown in (**b**) as scatter plots of the investigation ratio (time spent investigating the novel object / time spent investigating both objects). Performance was compared to chance (i.e., IR = 0.5) using single-sample, two-tailed t-tests. There were no significant differences (ns) between the groups. * *p* < 0.05

Shown in Fig. 1a are data on cognitive performance in the Barnes Maze following four months of HF/HSD for transgenic and wildtype rats. The bar graphs are the mean ± SD for the latency to enter the goal box. The scores are the pooled average of all four test periods. There were no significant differences between male and female *ApoE ε4* carriers; neither was there any significant differences between wildtype males and females. A one-way ANOVA with multiple comparisons found a significant difference between the female *ApoE ε4* and male wildtype (F = 3.355, *p* = 0.045). Shown in Fig. 1b are data from NOP. None of the four conditions were better than chance (50%) on time spent with the novel object.

### Functional connectivity

**Fig. 2:**
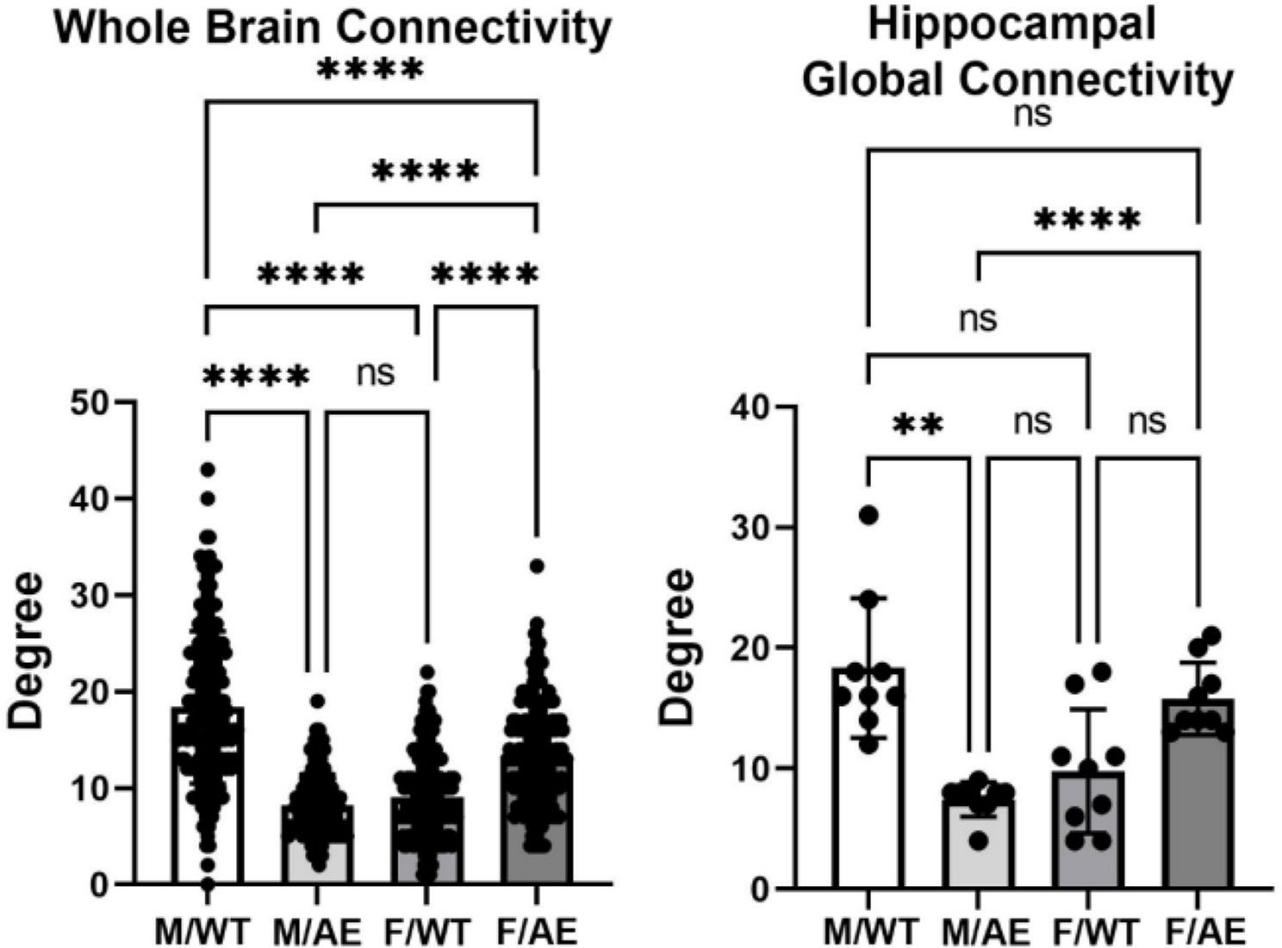
Global connectivity. Shown are bar graphs for the mean ± SD number of Degrees of functional connections for the whole brain and hippocampus together with a scatter plot of all brain areas represented in each. Male wildtype (M/WT) was significantly greater than all other experimental groups while female *ApoE ε4* (F/e4) was significantly greater than male *ApoE ε4* (M/e4) and female wildtype (F/WT). The M/WT global network density was 0.111 and the global average degree 18.383. The M/e4 global network density was 0.049 and the average degree 8.216. The F/WT network density was 0.055 and the average degree 9.114. The F/e4 network density was 0.080 and average degree 13.341. ** *p* < 0.01; **** *p* < 0.0001. Brain areas comprising the whole brain and hippocampus can be found in the Supplementary Excel File S2

Global brain connectivity as expressed in Degrees or number of connections (mean ± SD) for each of the experimental conditions is shown in Fig. 2. There was a significant main effect for genotype (two-way ANOVA, F_(3,498)_ = 166.1; *p* < 0.0001). Tukey’s post hoc multiple comparison test showed male wildtype on HFD/HSD was significantly greater than all other experimental conditions (*p* < 0.0001). Female *ApoE ε4* presented with the next highest number of Degrees that was significantly greater than male *ApoE ε4* and female wildtype (*p* < 0.0001). There was no significant difference between male *ApoE ε4* and female wildtype. Global brain hippocampal connectivity is shown on Fig. 2. There was a significant main effect for genotype (two-way ANOVA, F_(3,24)_ = 13.61; *p* < 0.0001). The male wildtype were significantly greater than male *ApoE ε4* (*p* < 0.0001) and female wildtype (< 0.01), while female *ApoE ε4* were significantly greater than male *ApoE ε4* (*p* < 0.01) and female wildtype (*p* < 0.05). Given the hyperconnectivity of the male wildtype on HFD/HSD as compared to the other experimental conditions we analyzed the connectivity in the context of feeding behavior.

Table 1 is a list of brain areas taken from recent review by Alcantara et al. [29], on the neural circuits governing the different phases of feeding.

**Table 1:** Degrees for brain areas forming the neural circuit regulating feeding and metabolism. Degrees of connectivity

Brains areas involved in the three phases of eating behavior: procurement, consumption and termination				
Brain areas	Male WT	Male APOE4	Female WT	Female APOE4
Accumbens core	15	7	7	11
Accumbens shell	20	14	7	16
Arcuate nucleus	6	4	3	4
Bed nucleus stria terminalis	27	5	11	9
Central amygdaloid nucleus	13	7	9	7
Dorsal raphe	13	6	7	20
Insular ctx	19	5	5	8
Lateral hypothalamus	22	11	16	17
Locus coeruleus	23	6	9	10
Intermediate cerebellar nucleus	32	12	7	13
Lateral cerebellar nucleus	29	6	6	9
Medial cerebellar nucleus fastigial	34	9	10	11
Parabrachial nucleus	33	13	13	21
Paraventricular nucleus hypothalamus	19	11	15	11
Paraventricular nucleus thalamus	4	7	7	11
Periaqueductal gray thalamus	29	9	7	22
Substantia innominata	25	8	13	10
Ventral tegmental area	18	9	12	15
Zona incerta	15	6	17	16

**Fig. 3:**
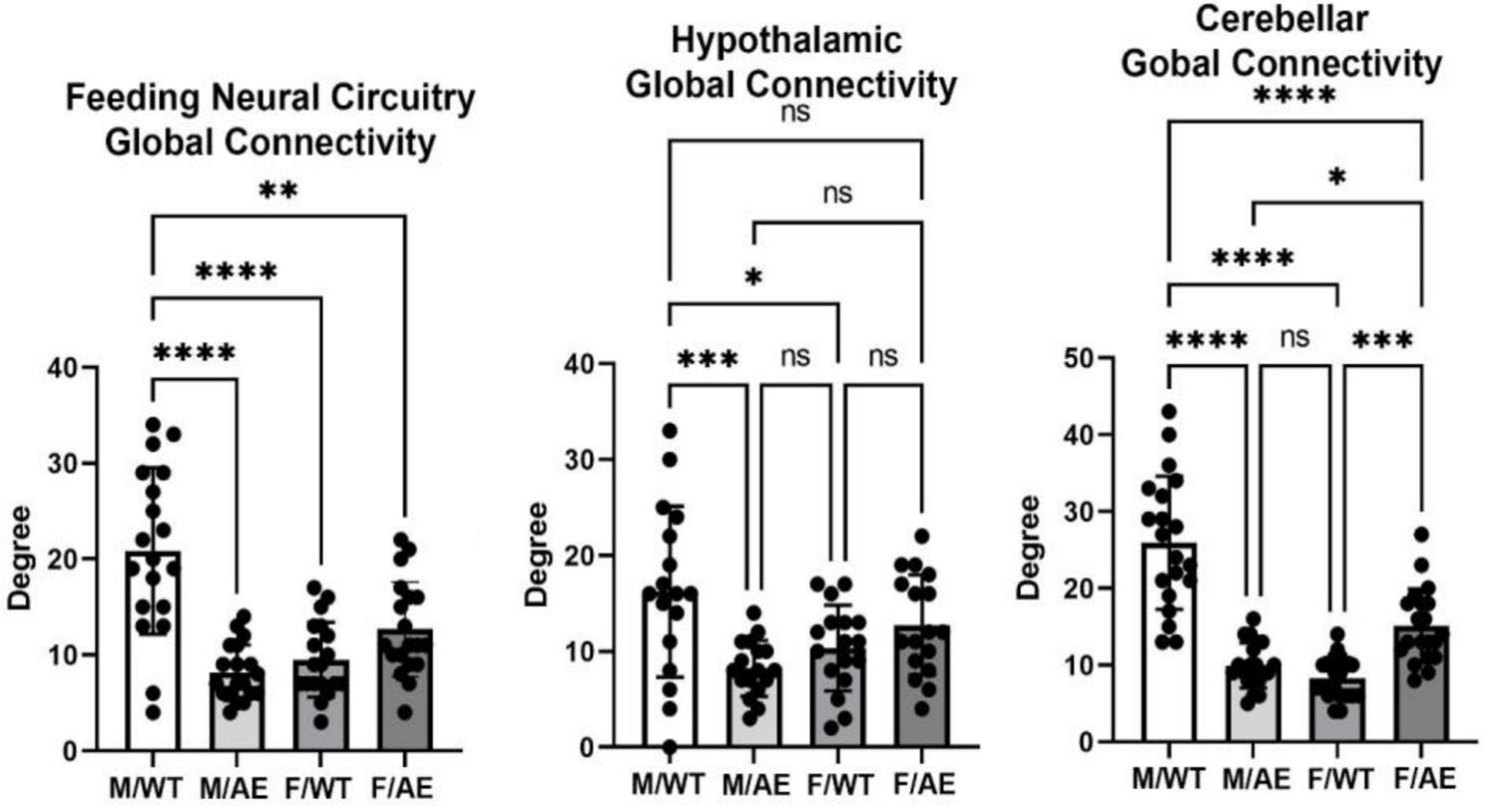
Global connectivity around feeding. Shown are bar graphs for the mean ± SD number of Degrees of functional connections for the feeding neural circuit, hypothalamus and cerebellum together with a scatter plot of all brain areas represented in each. Labels are the same as Fig. 2. * *p* < 0.05; ** *p* < 0.01; *** *p* < 0.001; **** *p* < 0.0001. Brain areas comprising the feeding circuit, hypothalamus and cerebellum can be found in the Supplementary Excel File S2

The global connectivity for this neural circuit for each of the experimental conditions (mean ± SD) is shown in Fig. 3. There was a significant main effect for genotype (two-way ANOVA, F_(3,51)_ = 21.53; *p* < 0.0001). Tukey’s post hoc test showed male wildtype on HFD/HSD was significantly greater than male *ApoE ε4* (*p* < 0.0001), female wildtype (< 0.0001), and (*p* < 0.01), while female *ApoE ε4* was significantly great than male *ApoE ε4* (*p* < 0.01). A key region of the brain controlling feeding behavior is the hypothalamus shown in Fig. 3. Again, there was a main effect for genotype (F_(3,45)_ = 11.08; *p* < 0.0001). Post hoc tests showed male wildtype was significantly greater than male *ApoE ε4* (*p* < 0.0001), females wildtype (*p* < 0.01) and female *ApoE ε4* (*p* < 0.05), while female *ApoE ε4* was significantly greater than male *ApoE ε4* (*p* < 0.05). The cerebellum and the deep cerebellar nuclei have also been identified as contributing to the organization of feeding behavior and metabolism (Fig. 3). There is a main effect for genotype (F_(3,57)_ = 52.30; *p* < 0.0001) with male wildtype connectivity is significantly (*p* < 0.0001) greater than the other experimental conditions.

**Fig. 4:**
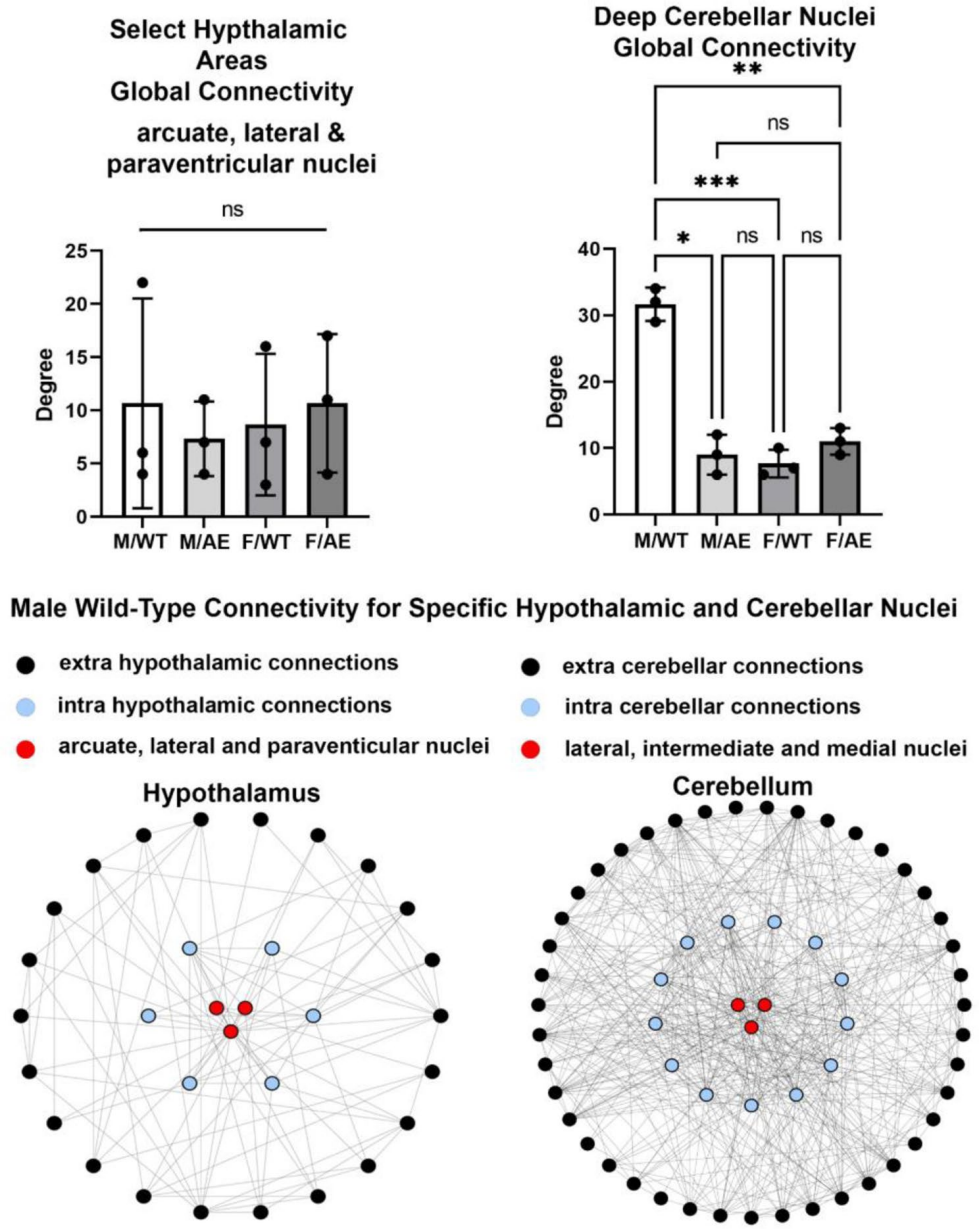
Global connectivity to select feeding nuclei. Shown are bar graphs for the mean ± SD for the number of Degrees of functional connections for the three hypothalamic and cerebellar nuclei found to be key in regulating feeding behavior in rodents together with a scatter plot of their individual values. A network maps for the male wildtype (M/WT) of each is shown below. The three red circles represent the three brain areas from each brain region. The blue circles are the intra or within connections from these three nodes to their respective brain region, i.e. hypothalamus and cerebellum. The outer black dots are the extra or outside connections from these nodes to other brain areas. Nonsignificant (ns); * *p* < 0.05; ** *p* < 0.01; *** *p* < 0.001

The arcuate, lateral, and paraventricular nuclei of the hypothalamus and the deep cerebellar nuclei have a major role in controlling feeding [29]. The connectivity of these three hypothalamic and cerebellar nuclei is shown in Fig. 4. The hypothalamic connectivity in male wildtype are not significantly different from the other experimental groups. In contrast the connectivity of the deep cerebellar nuclei are significantly greater than the other experimental groups. The intra and extra hypothalamic and cerebellar connectivity can be visualized by the network maps below the bar graphs. The circles are nodes, and the lines are edges. The inner blue nodes are the functional connections of the different nuclei to their immediate surroundings i.e. either other hypothalamic or cerebellar nuclei. The black nodes are functional connections to other brain areas.

## Discussion

The present study examined the intersection of both aging and diet as risk factors for AD with both male and female wildtype and *ApoE ε4* knock-in rats after four months on HFD/HSD or “Western” diet. At four months of age, all rats were put on the HFD/HSD diet ad libitum. Four months later a period equivalent to ca. 8.7 human years [30] they were tested for cognitive behaviors and imaged for differences in resting state BOLD functional connectivity (rsFC). To our surprise male and female *ApoE ε4* carriers were unaffected by the HFD/HSD diet as compared to wild-type rats; instead, it was the male wild-type, but not the female that showed behavioral and neuroradiological changes. These finding are discussed below with respect to preclinical and clinical studies on the behavior and neurobiology of *ApoE ε4* carriers and the influence of diet.

### Behavior

Eight-month-old male wildtype rats on HFD/HSD showed a modest deficit in cognitive function when evaluated in the Barnes maze while the male and female *ApoE ε4* carriers and female wildtype showed no deficits. In a previous study using the same genetic rat model we reported 6-month-old human *ApoE ε4* knock-in rats feed a normal diet were more likely to have cognitive and behavioral problems as compared to female carriers early in life [24]. In the same rat model, we reported 8-month-old female carriers on normal diet show enhanced microvascular density in the hippocampus hypothesized to be a neuroadaptive response helping to spare cognitive function [23]. These and other studies on rodent models of *ApoE ε4* show genotype-specific changes in behavior and brain structure characteristics of early neurodegenerative pathology [22, 31–34]. Given the risk associated with HFD/HSD for cardiovascular disease, Alzheimer’s disease, and early dementia, we hypothesized *ApoE ε4* male and female rats would show exacerbated signs of cognitive dysfunction. Instead, *ApoE ε4* male and female rats were spared the anticipated negative effects of the HFD/HSD, while wildtype males, but not females showed the most changes. Was the HFD/HSD protective in these 8-month-old *ApoE ε4* rats, evidence of antagonistic pleiotropy, i.e., genes that provide resilience for developmental maturation and reproduction but are detrimental in old age [35]? Indeed, Janssen et al., reported female *ApoE ε4* mice feed HFD/HSD showed no deficits in spatial learning together with a reduction in neuroinflammation in the hippocampus as compared to wildtype controls on HFD/HSD [36]. In a recent review on feeding behavior and metabolism, Alcantara and colleagues argue that evolution has driven a neurobiology that favors the overconsumption of calories during times of plenty to offset the dearth of calories during times of famine [29]. *ApoE ε4’s* role in lipid metabolism, transport of cholesterol and complex lipids needed in neural development, membrane maintenance, and repair would be essential in handling the lipid load in HFD/HSD [11]. Jones et al., studied the behavioral and metabolic changes in male and female human *ApoE ε3* and *ApoE ε4* knock-in mice fed HFD/HSD as compared to low fat diet and reported male *ApoE ε4* mice were more susceptible to metabolic disturbances but not female *ApoE ε3* and *ApoE ε4* mice [21]. This is in contrast to our data that showed male *ApoE ε4* were unaffected by the HFD/HSD. The metabolic sensitivity of the male *ApoE ε4* mice to HFD/HSD but not female *ApoE ε4* mice was corroborated by Matter et al., with the added distinction that females showed impairment in cognitive function [20]. In a subsequent study, Jones, and coworkers feed *ApoE ε3* and *ApoE ε4* mice HFD/HSD for 12 weeks and found the *ApoE ε3* genotype but not *ApoE ε4* presented with significant neuroinflammation in the hippocampus as measured by activated microglia and astrocytes [37]. Interestingly, they argued the neuroinflammation was neuroprotective in the *ApoE ε3* mice creating a neuroadaptive response to the HFD/HSD that would be beneficial in the future. Unfortunately, in our studies on *ApoE ε4* rats we did not do postmortem histology looking for evidence of gliosis and neuroinflammation.

### Functional connectivity - neural circuitry of feeding and metabolism

Male wildtype showed whole brain hyperconnectivity as compared to the male carrier while the female *ApoE ε4* showed greater connectivity as compared to the female wildtype. The hyperconnectivity for the male wildtype on HFD/HSD was unexpected. However, in a previous study we reported female *ApoE ε4* rats at six months of age show hyperconnectivity in amygdala, paraventricular nucleus of the hypothalamus and ascending reticular activating system [24]. In this study the male wildtype and female carrier both showed greater global hippocampal connectivity as compared to the male *ApoE ε4* carrier. As noted above this connectivity was not reflected in any change in cognitive function for females but may be associated with the deficit in learning for the male wildtype. In the latter case, the hyperconnectivity may reflect neuroplastic compensation in response to the early stages of brain injury and loss of function [38, 39].

The sensitivity of the male wildtype to the HFD/HSD as measured by alterations in cognitive function and functional connectivity suggested the risk factor was not *ApoE ε4*, but instead, a metabolic disorder. While body weights were only recorded through the first 8–9 weeks of HFD/HSD there were no significant changes between wildtype and carriers for either sex (Supplementary Fig S1). So obesity was not a contributing factor. Metabolism involves a balance in appetite and energy utilization. In a recent review conducted by Alcantara et al., a model was proposed that describes the interconnected neural circuitry responsible for regulating the different phases of feeding, including appetitive, consummatory, and terminating phases [29]. The circuits that control feeding are complex and involve multiple regions of the brain, most notably the hypothalamus. Within the hypothalamus, various nuclei host distinct populations of neurons that have significant roles in controlling appetite and energy balance [40–42]. These include neurons that respond to or produce orexigenic peptides such as ghrelin [41], and neuropeptide Y [43], and those that respond to or produce anorexigenic signaling molecules e.g. leptin [44] and melanocortin [45]. More specifically, the arcuate nucleus is home to a specific group of neurons known as agouti-related peptide (agRP) neurons in the arcuate nucleus that play a central role [46] in regulating all aspects of feeding in coordination with other hypothalamic areas [29, 47].

When the many brain areas controlling feeding (Table 1) are combined as a single hub it was the wild-type males on HFD/HSD that showed a significant global increase in connectivity exceeding all other experimental conditions. The global connectivity of the hypothalamus as a single node is also extensive in wildtype males and significantly greater than the other experimental conditions. However, the global connectivity of the three key hypothalamic areas i.e., acuate, lateral, and paraventricular nuclei, noted for their role in controlling the distinct phases of feeding present with only a modest number of connections and are not significantly different between experimental groups. A seminal paper by Low and colleagues identified neurons in the cerebellar nuclei, which are characterized by their unique molecular and topographical properties, were found to be activated in response to feeding and nutrient infusion resulting a decrease in food intake [48]. Activation of the deep cerebellar nuclei terminates feeding by altering dopaminergic signaling to the striatum. Our study looking at the interaction between genes and diet spotlighted these cerebellar nuclei e.g. lateral (dentate) intermediate (interposed) and medial (fastigial) and analyzed their connectivity. Indeed these cerebellar nuclei had an extensive network of connections far exceeding the hypothalamic nuclei but only in the male wildtype rats. How this hyperconnectivity is affecting feeding and metabolism in male wildtype is unknown.

### Limitations

The study was not designed to follow feeding and metabolism, points of interest only raised after analyzing the connectivity data. Circadian measures of feeding, metabolism, blood and urine chemistry for each experimental group would have enhanced our understanding of the effect of HFD/HSD on wild type and *ApoE ε4 carriers* and why this genetic rat model is resilient - an example of antagonistic pleiotropy? Equally important would have been the same four experimental groups but maintained on normal rat chow. Unfortunately the study had only a limited number of *ApoE ε4* rats preventing the study of controls on a normal diet. Hence all comparisons are with respect to HFD/HSD. What would be typical behavior or connectivity is uncertain. As noted above, there was no post-mortem histology to confirm the presence or absence of neuroinflammation as reported by Jones et al., [21]. Still another limitation was the collection of functional connectivity data under light isoflurane anesthesia to minimize motion and physiological stress [49]. What constitutes a “resting state” condition in a head restrained, awake rodent could be debated. The administration of anesthesia can decrease the strength of the BOLD signal; however, it does not disrupt the connectivity between brain regions. This has been observed in various species and under different physiological conditions [50–54].

## Summary

The present study examined the effect of high fat, high sugar diet on male and female, wildtype and *ApoE ε4* knock-in rats with the expectation that carriers would present with deficits in cognition and functional connectivity. The results were unexpected. The genetic risk was overshadowed by the diet. Male wildtype rats were most sensitive to the HFD/HSD presenting with a deficit in cognitive performance and enhanced functional connectivity in neural circuitry associated with food consumption and metabolism. The deep cerebellar nuclei key in the regulation of feeding behavior showed hyperconnectivity in male wildtype but not in female wildtype or female and male *ApoE ε4* rats.

## Electronic supplementary material

Below is the link to the electronic supplementary material.

**Supplementary Material 1: Fig. S1.** Body weights

**Supplementary Material 2:** Brain areas

## Data Availability

No datasets were generated or analysed during the current study.

## Declarations

### Author information

Colarusso, Yeboah, and Gupta responsible for data acquisition, Ortiz, Chang, Kulkarni responsible for data analysis, Ferris manuscript preparation.
