# Supplementary figures and images for: APOE4 rat model of Alzheimer’s disease: sex differences, genetic risk and diet
Source: BMC Neurosci. 2024 Nov 6;25:57. doi: 10.1186/s12868-024-00901-z (PMC11539573; doi:10.1186/s12868-024-00901-z)

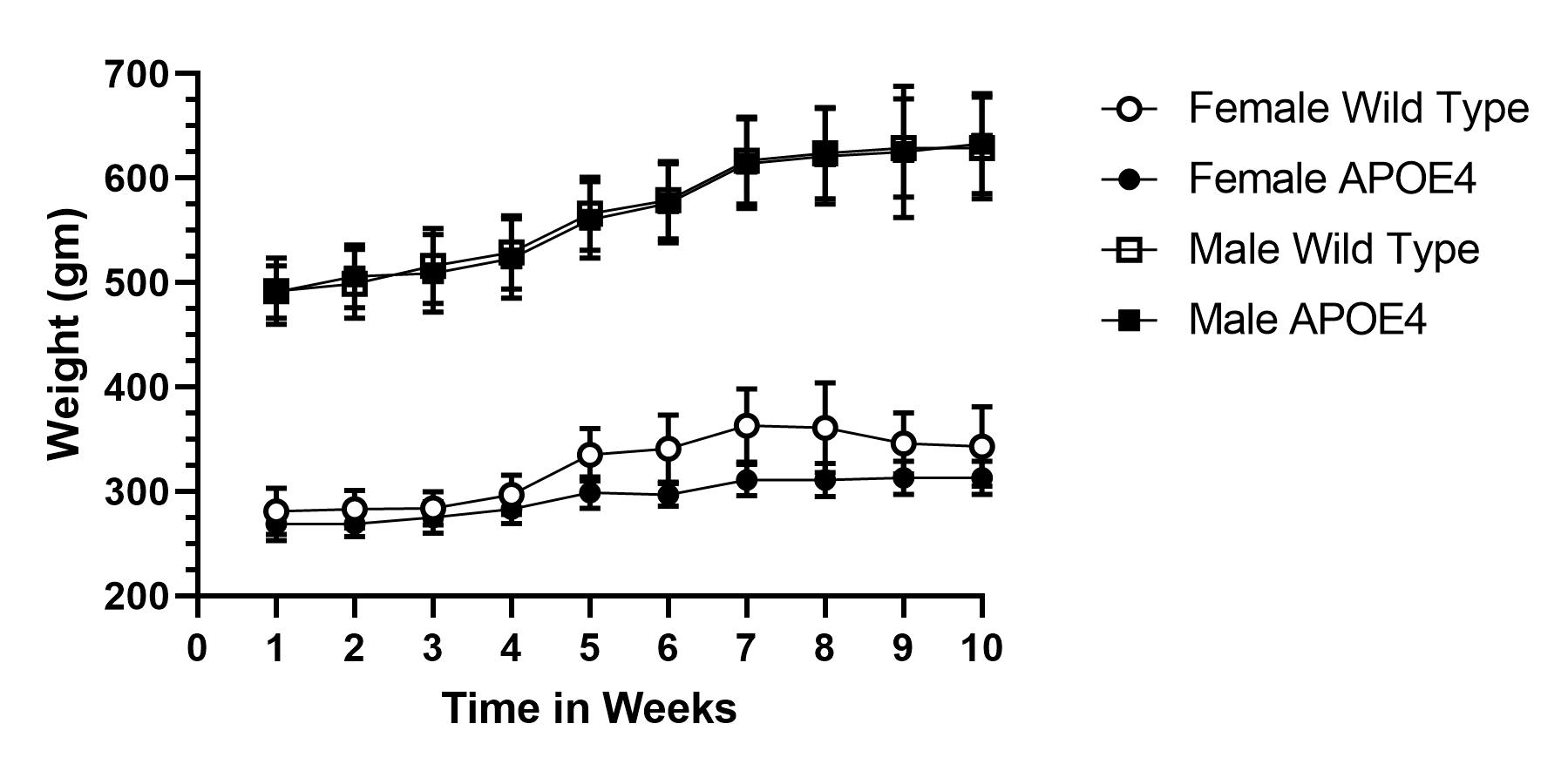

Supplement: Supplementary file 1 — Supplementary Material 1: Fig. S1. Body weights [file 12868_2024_901_MOESM1_ESM.jpg]
